# Metagenomic Next-Generation Sequencing for the Diagnosis of Neonatal Infectious Diseases

**DOI:** 10.1128/spectrum.01195-22

**Published:** 2022-11-21

**Authors:** Lu Chen, Yujuan Zhao, Jiakai Wei, Wendi Huang, Ying Ma, Xuefeng Yang, Yang Liu, Jing Wang, Han Xia, Zheng Lou

**Affiliations:** a Xi’an Children’s Hospital, Xi’an, China; b Neonatal Intensive Care Department, Xi’an Children’s Hospital, Xi’an, China; c Department of Scientific Affairs, Hugobiotech Co., Ltd., Beijing, China; Hôpital Saint-Louis

**Keywords:** neonate, mNGS, infection, the best timing, antimicrobial therapy

## Abstract

Infectious diseases pose a fatal risk to neonates. Timely and accurate pathogen detection is crucial for proper clinical diagnosis and therapeutic strategies. Limited sample volumes from neonatal patients seriously hindered the accurate detection of pathogens. Here, we unravel that metagenomic next-generation sequencing (mNGS) of cell-free DNA (cfDNA) and RNA can achieve unbiased detection of trace pathogens from different kinds of body fluid samples and blood samples. We enrolled 168 neonatal patients with suspected infections from whom blood samples (*n *= 153), cerebrospinal fluid samples (*n *= 127), and respiratory tract samples (RTSs) (including bronchoalveolar lavage fluids, sputa, and respiratory secretions) (*n *= 51) were collected and analyzed using mNGS. High rates of positivity (70.2%; 118/168) of mNGS were observed, and the coincidence rate against the final clinical diagnosis in positive mNGS cases reached 68.6% (81/118). The most common causative pathogens were Klebsiella pneumoniae (*n *= 12), Escherichia coli (*n *= 12), and Streptococcus pneumoniae (*n *= 8). mNGS using cfDNA and RNA can identify microbes that cannot be detected by conventional methods in different body fluid and blood samples, and more than 50% of these microbes were identified as causative pathogens. Further local polynomial regression fitting analysis revealed that the best timing for mNGS detection ranged from 1 to 3 days after the start of continuous antimicrobial therapy. Diagnosed and guided by mNGS results, the therapeutic regimens for 86 out of 117 neonatal patients were changed, most of whom (80/86) completely recovered and were discharged, while 44 out of 86 patients completely or partially stopped unnecessary medication. Our findings highlight the importance of mNGS in detecting causative DNA and RNA pathogens in infected neonatal patients.

**IMPORTANCE** To the best of our knowledge, this is the first report on evaluating the performance of mNGS using cfDNA and RNA from body fluid and blood samples for diagnosing neonatal infections. mNGS of RNA and cfDNA can achieve the unbiased detection and identification of trace pathogens from different kinds of neonatal body fluid and blood samples with a high total coincidence rate (226/331; 68.3%) against final clinical diagnoses by sample. The best timing for mNGS detection in neonatal infections ranged from 1 to 3 days, rather than 0 days, after the start of continuous antimicrobial therapy. Our findings highlight the importance of mNGS in detecting causative DNA and RNA pathogens, and the extensive application of mNGS for the diagnosis of neonatal infections can be expected.

## INTRODUCTION

Infections, including sepsis, meningitis, and pneumonia (1, 2), are the primary cause of about 3 million global neonatal deaths per year (1, 3), which contributed more than 20% to neonatal deaths worldwide (4). Infected neonates are often treated empirically and unnecessarily (5). Besides death, unoptimistic prognoses may occur, including several neurodevelopmental complications (3) and long-term disabilities (6), if timely diagnosis and proper therapy are not executed. Pathogenic microorganisms encompass a wide range of bacteria, viruses, and fungi (3, 5, 7, 8). Accordingly, not only timely but also accurate identification of pathogens and appropriate therapies are needed to improve prognoses.

Blood, cerebrospinal fluid (CSF), and bronchoalveolar lavage fluid (BALF) are ideal samples for diagnosing neonatal infections (3, 7–10). However, more than 60% of pathogens cannot be detected in blood, CSF, or BALF culture (11–13). Some pathogens with negative blood cultures, such as Streptococcus pneumoniae, may result in higher mortality rates (14). Limited numbers of pathogens could be detected in a single experiment using hypothesis-based PCR (15) or antibody methods (16). With the first successful application of metagenomics next-generation sequencing (mNGS) in clinical infection of the central nervous system (CNS) (17), this unbiased technology has been extensively used to detect pathogens in various infections (18–20). Although the pathogen loads were higher in neonates (21) than in adults (22, 23), the volumes of samples obtained from neonates were lower than those obtained from adults (24), which seriously hindered the accurate detection of pathogens (25). The above-mentioned problems raised the question of how to accurately detect pathogens from neonatal samples with limited volumes using mNGS.

The accuracy of mNGS is determined by the ratio of the pathogen DNA concentration to the total DNA concentration in a sample (26), and a high ratio of human DNA to the total DNA can decrease the sensitivity of the assay (27). Differential lysis was used to filter human DNA (28). However, some viruses and bacteria, such as Pseudomonas aeruginosa, were vulnerable to DNA degradation caused by differential lysis, yielding false-negative results by subsequent mNGS (20, 28). mNGS using DNA obtained from direct pathogen lysis also lost some pathogens such as Mycobacterium bovis and Mycobacterium tuberculosis (29). Conversely, mNGS using cell-free DNA (cfDNA) has been proven to be a promising tool for the detection of pathogens from body fluids, with higher sensitivities (75 to 91%) than conventional methods (20). For adults, we also found that mNGS using cfDNA can accurately detect trace pathogens from 200-μL CSF samples (30), and the sensitivity for the testing of BALF samples can reach about 90% (19). Hence, we hypothesized that mNGS using cfDNA can accurately detect pathogens from limited-volume samples in neonatal infections.

In the present study, we collected blood, CSF, and respiratory samples from neonates with suspected infections to test the accuracy of mNGS using cfDNA and RNA against conventional methods and clinical diagnoses. A fitting model was constructed to evaluate the effects of empirical therapy on pathogen detection by mNGS and conventional methods and determine the best timing for mNGS testing. Besides, changes in medication according to the mNGS results are also summarized.

## RESULTS

### Clinical characteristics.

A total of 168 neonates with suspected infections were enrolled. These 102 males and 66 females were born with a median birth weight of 3,115 g and a median gestational age of 38 weeks 5 days (38+5 weeks), and the age upon admission ranged from 1.3 h to 120 days (Table 1). The main clinical symptoms included fever (*n *= 98), apnea or respiratory distress (*n *= 53), dystonia (*n *= 51), gastrointestinal dysfunction (*n *= 25), tachycardia (*n *= 24), convulsion (*n *= 11), consciousness disorder (*n *= 11), and coma (*n* = 9) (Table 1).

**TABLE 1:** Physical information and clinical characteristics of the enrolled neonatal patients

Parameter	Value
Physical information	
No. of patients of sex	
Male	102
Female	66
No. of patients with route of delivery	
Cesarean section	93
Vaginal delivery	75
Median age (days) (IQR [min, max]; CI)	15 (19 [0.054, 120]; ~16–22)
Median gestational age (wks) (IQR [min, max]; CI)	38 + 5 (3 [27 + 3, 41 + 5]; 37 + 3–38 + 3)
Median birth wt (g) (IQR [min, max]; CI)	3,115 (672.5 [790, 4,300]; ~2,844.7–3,064)
No. of patients with clinical characteristic (%)	
Fever	98 (58.33)
Consciousness disorders	11 (6.55)
Seizure	11 (6.55)
Erythra or rash	12 (7.14)
Icterus	72 (42.86)
Groan	29 (17.26)
Irritation	26 (15.48)
Increased anterior fontanelle tension	2 (1.19)
Gastrointestinal dysfunction	25 (14.88)
Bradypnea/tachypnea	53 (31.55)
Tachycardia	24 (14.29)
Shock	9 (5.36)
Intracranial hemorrhage	33 (19.64)
Hydrocephalus	6 (3.57)
Dystonia	51 (30.36)

**FIG 1:**
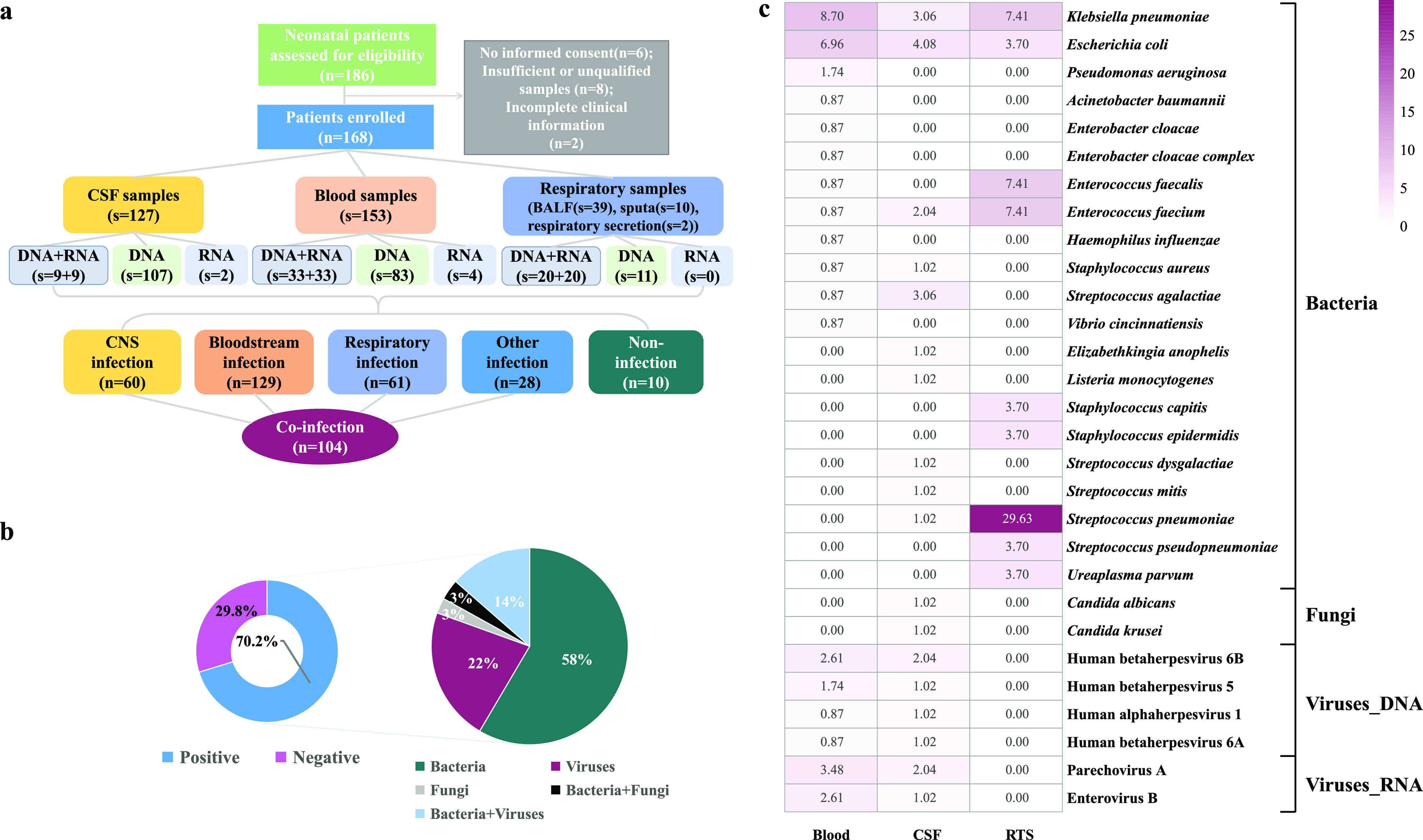
Flowchart and pathogen distribution. (a) Participant selection flow diagram. (b) Composition of microbes identified by mNGS. (c) Causative pathogens identified from different types of samples by mNGS. CNS, central nervous system; CSF, cerebrospinal fluid; RTS, respiratory tract sample; n, number of neonates; s, number of samples.

For further diagnoses, 331 samples were collected from 168 neonates for mNGS, including 153 blood samples (116 cfDNA and 37 RNA tests), 127 CSF samples (116 cfDNA and 11 RNA tests), and 51 respiratory tract samples (RTSs) (39 BALF, 10 sputum, and 2 respiratory secretion samples) (31 cfDNA and 20 RNA tests). A total of 158 neonates were diagnosed as having infections, including bloodstream (129), CNS (60), respiratory tract (61), urinary system (9), and other (19 skin and soft tissue, umbilical cord, appendix, peritoneum, intestine, and bone marrow) infections (Fig. 1a). Besides, 104 neonates had multifocal infections, including CNS-bloodstream (*n *= 41) and respiratory tract-bloodstream (*n *= 30) infections (see Table S1 in the supplemental material).

### Pathogen profiles.

A high positivity rate (70.2%; 118 out of 168 neonatal patients) (Fig. 1b) of mNGS was observed, and 66 underlying pathogens were identified from 118 patients, including 2 RNA viruses. Potential pathogenic bacteria, viruses, and fungi were found in 75.4% (89/118), 35.6% (42/118), and 5.9% (7/118) of neonatal patients, respectively, while coinfections were observed in 16.9% (20/118) of neonatal patients (bacterium-virus coinfections, 80%; bacterium-fungus coinfections, 20%) (Fig. 1b). The positive coincidence rate of mNGS was 68.6% (81 out of 118 neonatal patients).

On the whole, the most common causative pathogens revealed by mNGS were Klebsiella pneumoniae (*n *= 12), Escherichia coli (*n *= 12), and Streptococcus pneumoniae (*n *= 8) (Fig. S1). However, significant differences in the top causative DNA pathogens were found among different kinds of samples (Fig. 1c). For blood samples, the dominant pathogens were K. pneumoniae (8.7%; 10 out of 115 neonatal patients) and E. coli (6.96%; 8/115). The most commonly detected pathogens in CSF samples were E. coli (4.08%; 4/98), K. pneumoniae (3.06%; 3/98), and Streptococcus agalactiae (3.06%; 3/98), while S. pneumoniae (29.63%; 8/27) was the dominant pathogen in RTSs. For RNA viruses, we detected only parechovirus A (4 cases) and enterovirus B (4 cases) (Fig. S1). Besides, the presence of some pathogens mentioned above was successfully confirmed by quantitative PCR (qPCR), PCR, or Sanger sequencing (Table S2).

### Advantages of mNGS over conventional methods.

**FIG 2:**
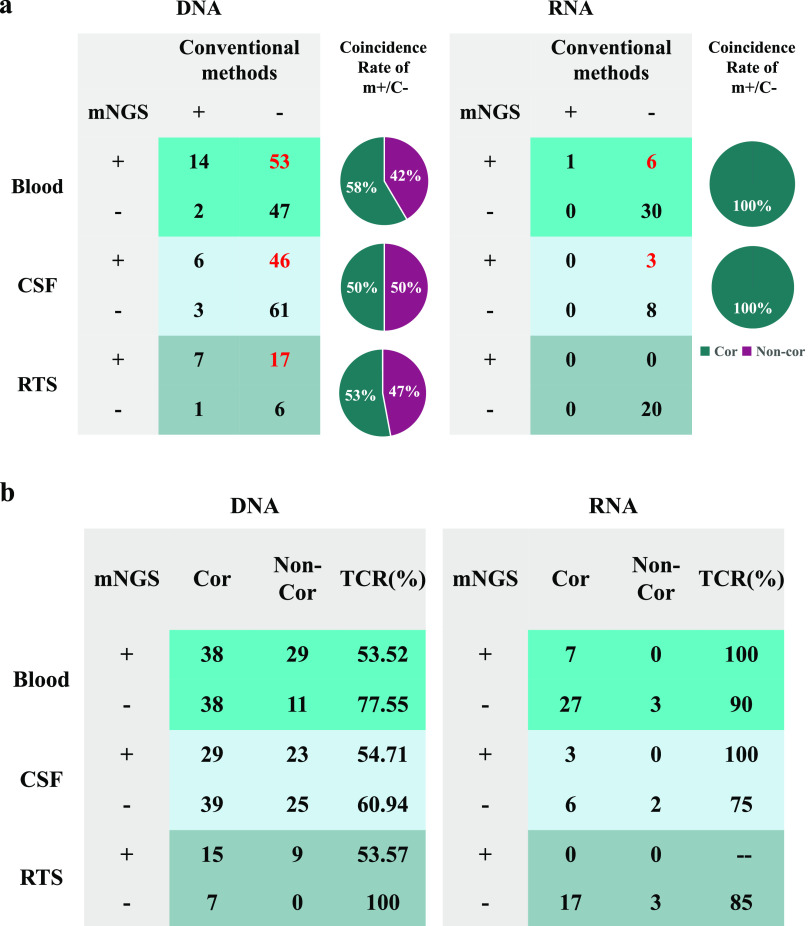
mNGS performance and coincidence rate. (a) Advantages of mNGS over conventional methods. For the samples with positive mNGS (m+) and negative conventional method (C−) results, we further calculated the clinical coincidence rate, and the pie charts show that 50% to 100% of the mNGS results were consistent with the final clinical diagnoses. (b) Agreement of mNGS results with clinical diagnoses. CSF, cerebrospinal fluid; RTS, respiratory tract sample; Cor, coincidence with the final clinical diagnosis; Non-Cor, noncoincidence with the final clinical diagnosis; TCR, total coincidence rate.

Compared with conventional testing methods (CTMs), mNGS demonstrated a better performance in identifying pathogens from blood, CSF, BALF, and sputum samples. For blood samples, the positivity rate of mNGS using cfDNA was 57.8% (67/116), much higher than that of the CTM (13.8%; 16/116). Similar trends were found for CSF (mNGS, 44.8%; CTM, 7.8%), BALF (mNGS, 78.8%; CTM, 24.2%), and sputum (mNGS, 71.4%; CTM, 14.3%) samples. The sensitivities of mNGS using cfDNA from blood samples, CSF samples, and RTSs against conventional methods were 87.5%, 66.7%, and 87.5%, respectively. Besides, mNGS can identify microbes that cannot be detected using conventional methods (Fig. S1), and more than 50% of the microbes (blood, 58% [31/53]; CSF, 50% [23/46]; RTS, 53% [9/17]) were identified as being causative pathogens (Fig. 2a). The total coincidence rate (TCR) of mNGS using cfDNA was 63.1% (166/263) at the sample level (Fig. 2b).

mNGS also exhibited a better performance in detecting RNA viruses. The positivity rates of mNGS using blood and CSF samples were 18.9% (7/37) and 27.3% (3/11), respectively (Fig. 2a). An RNA virus was detected only in a blood sample of one case using antibody measurement. Meanwhile, all of the viruses detected using mNGS were identified as causative pathogens, and the negative coincidence rates of mNGS using RNA ranged from 75% to 90% (Fig. 2b). In general, the TCR of mNGS using cfDNA and RNA was 68.3% (226/331) at the sample level. The above-described results further indicate that mNGS using blood and body fluid samples may be preferred for the examination of neonatal infections by DNA or RNA pathogens.

### mNGS performance in CNS-bloodstream coinfections.

**FIG 3:**
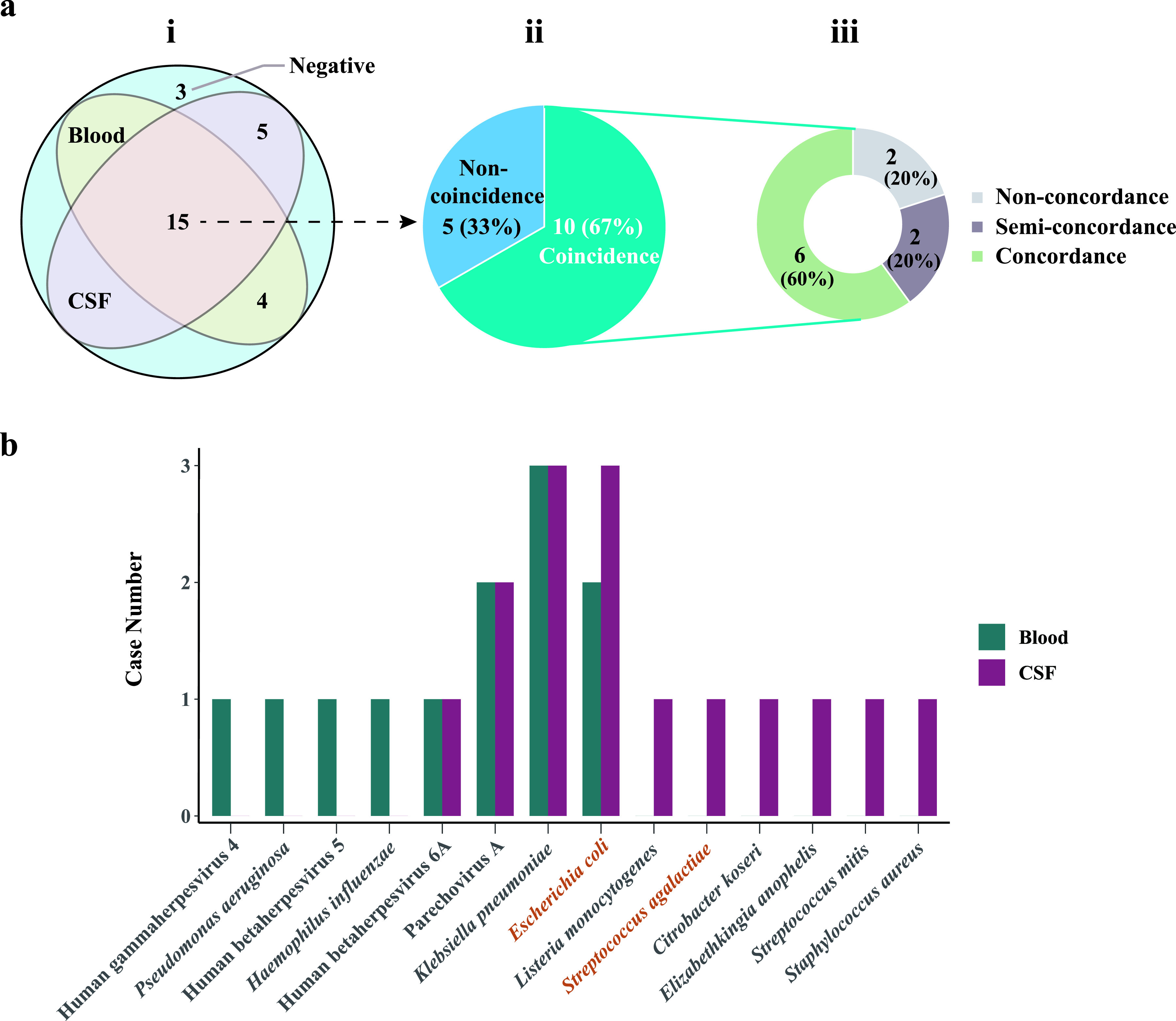
mNGS results for cerebrospinal fluid (CSF) and blood samples of neonates with suspected CNS-bloodstream infections. (a) Positivity rates of CSF and blood mNGS tests, TCRs of positive results from both CSF and blood samples, and concordance of pathogens identified in blood and CSF samples. Identical pathogens were identified from the blood and CSF samples of 8 neonates. Different pathogens were identified from the blood and CSF samples of 2 neonates. (b) Pathogen profiles detected in blood and CSF samples.

Blood and CSF samples were simultaneously collected from 63 neonates with suspected CNS-bloodstream coinfections for mNGS using cfDNA (*n* = 62) or RNA (*n* = 5). According to the final clinical diagnoses, 53 neonatal patients had a bloodstream infection, and 27 of them were diagnosed with a CNS-bloodstream coinfection. The microbes identified in the blood and CSF samples of each patient are listed in Table S3. The results of mNGS using both blood and CSF samples showed a positivity rate of 88.9% (24/27) in neonatal patients, which was better than the performance of mNGS using blood (19/27; 70.4%) or CSF (20/27; 74.1%) samples alone (Fig. 3a). The coincidence rate of mNGS using CSF against the final diagnosis (14/27; 51.9%) was higher than that using blood (10/27; 37.0%) (Table S3). Hence, mNGS testing of both blood and CSF samples may be considered the preferred test for neonates with suspected CNS-bloodstream coinfections.

Furthermore, the microbes detected from the blood and CSF samples of these 27 patients, considered to be coincident with the final diagnoses, were compared. The top causative pathogens (K. pneumoniae, E. coli, and parechovirus A) identified in the blood samples were the same as those identified in the CSF samples. However, there were some differences in the whole-pathogen profiles between the blood and CSF samples, such as S. agalactiae, human betaherpesvirus 5, and Listeria monocytogenes (Fig. 3b). The difference in the pathogen profiles raises two questions, whether there is a definite direction of pathogen spread and whether the spread is from the blood to the CSF or from the CSF to the blood in neonates with CNS-bloodstream coinfections, which needs further investigation.

### Reference for the timing of mNGS tests and treatment strategies.

**FIG 4:**
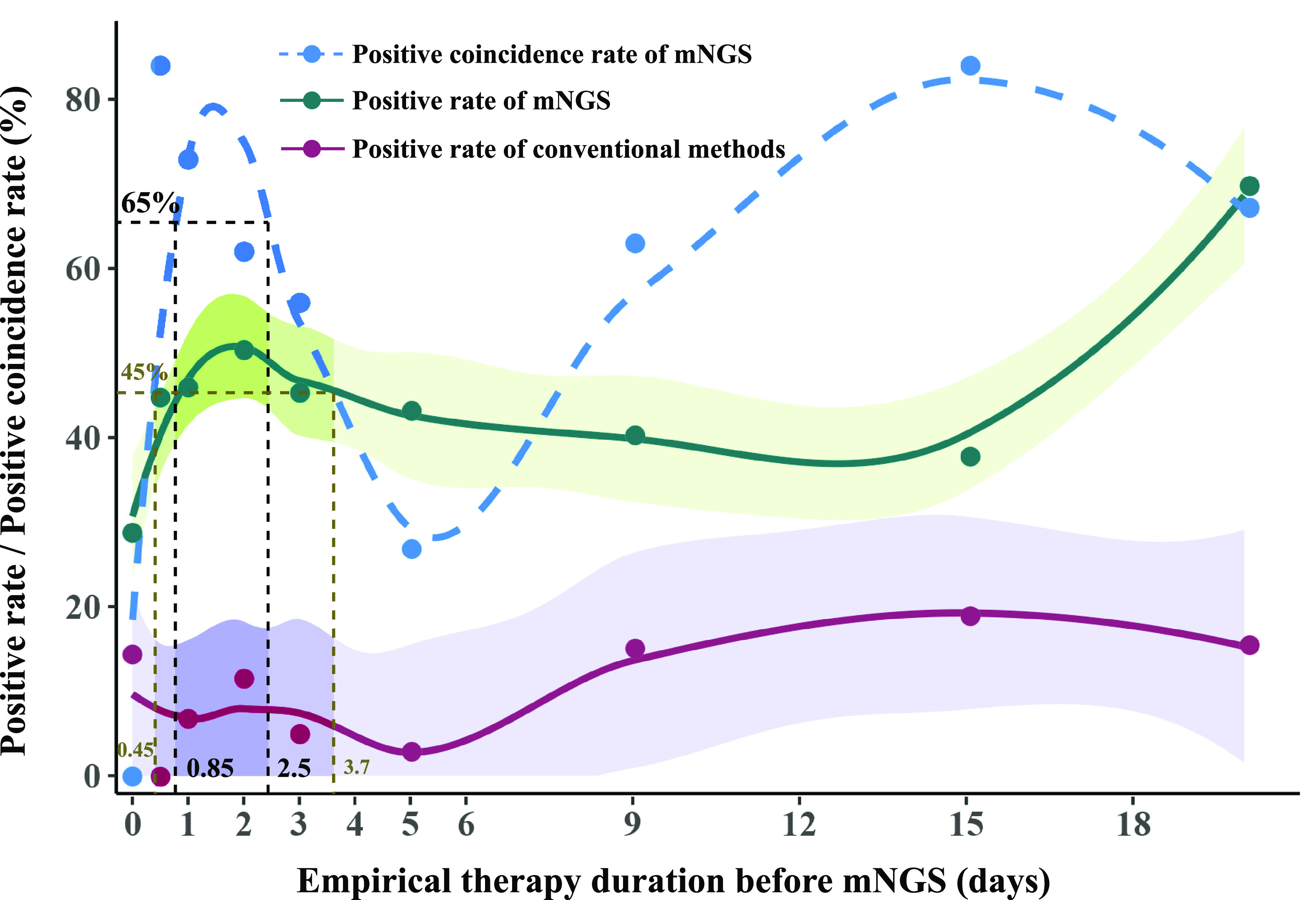
The positive coincidence rate and the positivity rate of mNGS changed with the duration of empirical therapy before mNGS tests. Green circles represent the positivity rates of mNGS with empirical therapy for 0 days, 0.5 days, 1 day, 2 days, 3 days, 4 to 5 days, 6 to 9 days, and 10 to 15 days. The green curve represents the positivity rates of the mNGS fit using local polynomial regression fitting. The purple circles and curve represent the positivity rates of conventional methods. Bands represent 95% CIs. The blue circles and dashed curve represent the fit positive coincidence rates of mNGS.

Almost all of the neonates (*n* = 164) enrolled in this study were treated with empirical therapy at the onset of clinical symptoms, and the duration before mNGS testing ranged from 0 to 55 days. The positive coincidence rate of mNGS, the positivity rate of mNGS, and positivity rate of conventional methods were fitted over the duration using local polynomial regression fitting (31) (Fig. 4). The fitting model showed that the positive coincidence rate (79.2%) peaked at a duration of 1.5 days, which reached 65% at durations of both 0.85 and 2.5 days. Besides, the positive coincidence rate was <45% at a duration of 4 days and was significantly decreased with duration. In general, the positivity rates of mNGS were much higher than those of conventional methods. Accordingly, we propose that mNGS testing should be performed at a duration of 1 to 3 days.

mNGS plays an important role in providing a reference for clinical therapy. Guided by mNGS results, the therapeutic regimens for most of the neonates (86/117) were changed, and more than half of these neonates, including 20 and 24 patients, completely and partially stopped unnecessary medication, respectively. Complete or partial changes in the types of drugs were carried out for 29 and 3 neonates, respectively, while 10 neonates were treated with additional drugs. Furthermore, empirical treatment regimens for 19 neonates were completely changed only according to mNGS results (Table 2). The accuracy and importance of mNGS in providing a reference for clinical therapy were confirmed by mNGS surveillance performed on seven critically ill neonatal patients after changing their treatment regimens (Fig. S2). Hence, the successful application of mNGS for the diagnosis and therapy of neonatal infections may play an important role in improving prognoses.

**TABLE 2:** Impact of mNGS on antimicrobial treatment of neonates with suspected infections

Modification	No. of patients (%)
Remove 1 agent	23 (19.7)
Remove 3 agents	1 (0.8)
Add 1 agent	8 (6.8)
Add 2 agents	2 (1.7)
Change completely	29 (24.8)
Change 1 agent	3 (2.6)
Stop	20 (17.1)
No change	31 (26.5)

## DISCUSSION

This is the first report on evaluating the performance of mNGS using cfDNA and RNA from body fluid and blood samples in the diagnosis of neonatal infections. Compared to conventional methods, mNGS exhibited better performances in both DNA and RNA tests, with a high TCR of 68.3%. The main causative pathogens were K. pneumoniae, E. coli, and S. pneumoniae in blood samples, CSF samples, and RTSs, respectively. Besides, mNGS testing of both blood and CSF samples should be considered the preferred examination for neonates with suspected CNS-bloodstream coinfections. Given the inevitability and indispensability of empirical therapy, the best timing for mNGS testing ranged from 1 to 3 days after the start of continuous antimicrobial therapy, and mNGS plays an important role in providing a reference for clinical therapy.

Our results confirmed that mNGS was superior to conventional methods for pathogen detection using blood and body fluid samples, with a high TCR (68.3%). First, the limited sample volumes (24) and the low detection efficiency of pathogen culture (16) hindered the comprehensive detection of pathogens using conventional methods. Second, the detection performance of conventional methods was sensitive to anti-infective therapy, whose effect on mNGS detection was hysteretic (12, 16). Besides, a high positivity rate (32) and a high sensitivity and specificity (20, 27) of mNGS using cfDNA were found for the detection of pathogens from both blood (32–34) and body fluid (20, 27, 29) samples. Our previous study showed that mNGS of cfDNA can detect trace pathogens from only 200 μL of CSF (reads per million [RPM] ranging from 2.28 to 226,781) (30), and the sensitivity of mNGS using cfDNA for the testing of BALF samples can reach 88.9%, much higher than that of mNGS using total DNA obtained by differential lysis (56.5%) (11). Accordingly, we propose that mNGS of cfDNA contributes to the diagnosis of neonatal infections to achieve a higher TCR, which is better than the performance of mNGS using DNA obtained by pathogen lysis (16).

The positive coincidence rate of mNGS changed with the duration of empirical therapy before mNGS tests. Initial empirical therapy can inhibit the cell wall synthesis of some pathogens or directly kill a portion of the pathogens, increasing cfDNA concentrations in the blood or body fluids in a short time (35). This may be why the positive coincidence rate of mNGS increased after initial empirical therapy, which was consistent with the results of a previous study (12). However, long and continuous antimicrobial therapy inhibited the growth of pathogens (36) and subsequently decreased the cfDNA concentrations. Hence, positivity rate of mNGS showed a downward trend from 2 days to 15 days after empirical therapy in our study. Zhang et al. (12) also proposed that antimicrobial treatment for >4 days in adults with CNS infection significantly affected detection by mNGS. Besides, the sudden rise in the positivity rate of mNGS after 15 days of empirical therapy may be attributed to the generation of drug-resistant pathogens and possible hospital-acquired infections, which may increase the cfDNA concentrations in the blood or body fluids. This dynamic process suggested that mNGS should be conducted with the right timing to decrease the time of therapy.

mNGS exhibited an important reference for treatment strategies. The value of mNGS as having a “rule-out” role (37, 38) is beneficial for minimizing the abuse of antimicrobial drugs (16). In our study, improved treatments according to mNGS results were implemented in most of the neonatal patients. For instance, before mNGS testing, prednisone and anti-infective therapy were administered to neonatal patient N5 with a suspected bacterial infection according to initially abnormal inflammatory indicators, while patient N5 was finally diagnosed with multisystem inflammatory disease by the negative mNGS result, and all of the antimicrobial drugs were discontinued in time.

Furthermore, reads of semiquantitative mNGS can reflect disease progression and treatment efficacy (12, 16, 39). In our study, after completing the specified antimicrobial treatment course for suppurative meningitis, neonatal patient N21 infected with E. coli was discharged based only on normal chemical indicators and negative culture results for a CSF sample, rather than performing mNGS, and antimicrobial drugs were also stopped. However, the neonatal patient was readmitted 2 days after discharge, from whom mNGS detected E. coli (RPM, 228,289) again. Subsequently, antimicrobial therapy was continuously performed, and mNGS detection was also conducted at different intervals of rehospitalization until no pathogen could be detected by mNGS. Finally, patient N21 was cured and discharged, without a recurrence of E. coli infection during follow-up. The above-described results emphasize the importance of mNGS in providing a reference for treatment strategies.

Most of the neonates (104/168) in this study were diagnosed with multiple-system coinfections. Some pathogens can spread over the body, such as S. pneumoniae from the upper respiratory tract through the bloodstream to other organs, including the meninges. Furthermore, the immature innate immune systems of neonates allow higher levels of bacteremia, enabling bacteria such as E. coli K1 and S. agalactiae to invade the CSF (40). In our study, for patients with CNS-bloodstream coinfections, E. coli was identified in both blood and CSF samples, while S. agalactiae was found only in CSF samples. The reasons for the inconsistency between the results of previous studies and our results may be as follows. The continuous use of antimicrobial drugs may result in a much lower load of S. agalactiae than that of E. coli in the blood, which may be caused by the difference in their growth rates (41, 42). Besides, less turnover of neonatal CSF might increase the concentration of S. agalactiae in the CSF (43). The above-described results emphasize the importance of mNGS testing using both blood and CSF for the diagnosis of neonatal CNS-bloodstream coinfections.

The dominant microbes identified by mNGS were K. pneumoniae, human betaherpesvirus 5, E. coli, and S. pneumoniae in this study. However, some microorganisms can be detected without being causative of symptoms, such as cytomegalovirus (CMV). We classified the detected CMV as a causative pathogen or an uncertain pathogen based on the presence or absence of organ lesions. It was reported previously that CMV can establish a latent infection in CD34^+^ hematopoietic progenitor cells of individuals with a competent immune system (44), and it was undetectable by the host immune system due to the low level of viral antigen (45). However, CMV reactivation can cause dissemination with severe morbidity and mortality due to weakened immune conditions (44, 46). Newborns with CMV infection, especially those with very low birth weight, may be at risk for hearing deficits and neurodevelopmental sequelae, even if they had no clinical diseases at birth (47–50). Given the high risk of reactivation and possible limitations in clinical diagnoses, we propose that clinicians should pay more attention to not only the causative pathogens but also these uncertain pathogens.

In addition, some microorganisms that are unlikely to cause disease in humans were also identified by mNGS in this study. Mupapillomavirus 2 and Dolosigranulum pigrum were classified as irrelevant pathogens (51, 52). Vibrio cincinnatiensis and Wickerhamomyces anomalus are rarely pathogenic, but it was also reported that they can cause infections (53–56), and W. anomalus was also detected by culture. Associated with the clinical manifestations in the patients, these two pathogens were classified as causative pathogens. The plant pathogen Xanthomonas campestris, which is a well-known contaminant in NGS studies (34), was not included in the analysis.

### Limitations.

There are some limitations of this study. First, mNGS tests of DNA or RNA were selectively performed in this study. Although after professional judgment and treatment by clinicians, most of the neonatal patients (158/168) recovered, incomplete detection might omit causative or latent pathogens, slightly prolong the treatment duration, increase the economic burden, and so on. Thus, we propose that mNGS testing of both DNA and RNA should be performed on neonatal patients if there is no limitation in funding. Second, given the challenge of collecting CSF and BALF samples from neonates, many more mNGS tests should be performed on neonatal patients with CNS-bloodstream coinfection or bloodstream-respiratory tract coinfection to investigate whether mNGS testing of blood samples can replace that of CSF or BALF samples, respectively. Third, as a control, data on the prognoses of neonates who were not diagnosed with reference to mNGS results should be collected to further unravel the important guidance of mNGS for the clinical treatment and prognoses of neonatal patients.

### Conclusions.

Our study emphasized that mNGS was overall superior to conventional methods for the detection of causative pathogens in infected neonatal patients. For neonates with suspected CNS-bloodstream coinfections, both blood and CSF samples should be considered for mNGS detection. The best timing for mNGS detection ranged from 1 to 3 days after the start of continuous therapy of antimicrobial drugs, and mNGS plays an important role in guiding clinical therapy. Our findings highlight the importance of mNGS in detecting causative DNA and RNA pathogens in infected neonatal patients.

## MATERIALS AND METHODS

### Ethics statement.

This study was reviewed and approved by the Ethical Review Committee of Xi’an Children’s Hospital (approval no. 20220001). All procedures were performed in strict compliance with the Regulations of Ethical Review of Biomedical Research Involving Human Subjects (2016), the Declaration of Helsinki, and the *International Ethical Guidelines for Biomedical Research Involving Human Subjects* (57).

### Study design.

From 1 January 2020 to 30 June 2021, neonatal patients with suspected infections of the CNS, bloodstream, respiratory tract, intestinal system, or urinary system admitted to the Neonatal Center, Xi’an Children’s Hospital, were enrolled for this prospective study. Patients with multiple-organ dysfunction syndrome, patients who required respiratory/circulatory support, or those who were born extremely preterm were admitted to the neonatal intensive care unit (NICU) for monitoring. The inclusion criteria were as follows. Suspected CNS infections were based on acute fever (>38.5°C) and one of the following signs: (i) stiff neck, (ii) consciousness disorders, (iii) increased anterior fontanelle tension, and (iv) meningeal irritation. Suspected respiratory infection was considered if the patient had new-onset shadow upon imaging examination and at least one of the following symptoms: (i) fever, (ii) cough, (iii) respiratory distress, and (iv) peripheral leukocytosis (>10 × 10^9^ cells/L) or leukopenia (<4 × 10^9^ cells/L). Patients with suspected bloodstream infections were enrolled according to sepsis diagnostic criteria (10). The exclusion criteria included the following: (i) the parents refused to sign informed consent, (ii) the samples were not sufficient for both mNGS and conventional testing or not qualified for sequencing, and (iii) the patients had incomplete clinical information.

After signing informed consent, samples were collected for tests. For suspected CNS infection, a CSF sample was collected, while a blood sample was collected if a lumbar puncture was refused. For suspected respiratory tract infections, the preferentially selected sample was a BALF sample, followed by sputum, respiratory secretion, and blood samples. Besides, blood samples were collected from patients with suspected infections of the bloodstream or other body systems. Based on the severity of the infection, multiple samples may have been collected from a patient at different intervals for mNGS detection and conventional diagnostic tests.

Given the limitations in funding, according to clinical characteristics, previous medication, and clinical experience, mNGS of cfDNA was the preferred examination, while for neonatal patients for whom DNA pathogen infections were definitely excluded (clinical manifestations and biochemical indicators suggested that viral infection could not be ruled out, but antibody measurements and PCR for common DNA pathogens were negative), mNGS of RNA was performed. Besides, for neonatal patients with indefinite clinical characteristics for discrimination or those with severe infections, mNGS testing of cfDNA and RNA was simultaneously conducted to avoid omitting pathogens (Fig. 1a). Conventional diagnostic tests used in this study included culture, antibody measurement, PCR, a (1,3)-β-d-glucan test (G test), and a galactomannan test (GM test). Physical information and clinical characterization data were also collected.

### Specimen processing and sequencing.

CSF samples and RTSs were collected into sealed sterile tubes and temporarily stored at temperatures below −20°C or transported to the laboratory of Hugobiotech (Beijing, China) on dry ice. Whole blood was collected into cell-free DNA blood collection tubes (BCT) (Streck, Inc., Omaha, NE, USA) and transported to the laboratory of Hugobiotech (Beijing, China) at 4°C within 24 to 48 h after sampling.

cfDNA was extracted and purified using the QIAamp DNA microkit (Qiagen, Hilden, Germany) according to the manufacturer’s instructions. We used a QIAamp viral RNA minikit (Qiagen, Hilden, Germany) to extract RNA, which was further transcribed in reverse using a Smart Moloney murine leukemia virus (MMLV) reverse transcriptase kit (TaKaRa Biotechnology Co., Ltd., Dalian, China). The concentration and quality of the extracts were tested using a Qubit 4.0 fluorometer (Thermo Fisher Scientific, MA, USA). DNA libraries and RNA libraries were constructed using a QIAseq ultralow-input library kit (Qiagen, Hilden, Germany) and a TruePrep DNA library prep kit (Vazyme, Jiangsu, China), respectively. The inspected library was sequenced on the NextSeq 550 platform (Illumina, San Diego, CA, USA); on average, 400 million reads can be generated from each chip. To ensure that enough data can be generated for analysis, no more than 20 samples were pooled on the same chip per run. On average, about 19 million reads were obtained for each sample.

### Bioinformatics analysis.

The obtained sequencing raw data were filtered to remove adapters and low-quality, low-complexity, and shorter reads (<35 bp). Human reads were removed by mapping to the human reference genome (hg38) using bowtie2 to obtain clean reads. Subsequently, using Burrows-Wheeler alignment (58), the obtained clean sequences were aligned with the Microbial Pan-genome Database, which was conducted according to the Reference Sequence Database of the National Center for Biotechnology Information (https://ftp.ncbi.nlm.nih.gov/genomes/). The lowest common ancestor method was used for annotation (18, 19).

In parallel with the samples, negative and positive controls were also set up for mNGS detection using the same procedure and bioinformatics analysis. The specific numbers of reads and reads per million (RPM) for each detected pathogen were calculated. For the detected bacteria, fungi, and parasites, a positive mNGS result was defined when the microorganism was not detected in the negative control (“no-template” control [NTC]) and when the genome coverage of the detected sequences belonging to this microorganism ranked among the top 10 of the same kind of microbes or when the ratio of the RPM of the sample (RPM_sample_) to the RPM of the NTC (RPM_NTC_) was >10 if the RPM_NTC_ was not equal to zero. For viruses, a positive mNGS result was considered when it was not detected in the NTC and at least 1 specific read was mapped to species or when the RPM_sample_/RPM_NTC_ ratio was >5 if the RPM_NTC_ was not equal to zero.

After being evaluated by the confidence coefficient and RPM, pathogens were defined according to whether the detected microbes were among the most commonly reported pathogens or whether the infections caused by the microbes were in accordance with the clinical features of the patients. Guided by the mNGS results, we would adjust the therapeutic regimens. For some detected microbes that could not be definitely considered causative pathogens, such as some viruses, we also summarized and mentioned them in our study to indicate their possibility of causing infection. Accordingly, we use the terms “causative pathogens” to represent the detected microbes that can be confirmed by clinical diagnosis and “uncertain pathogens” to represent the detected microbes that cannot be proven to be causal agents but also cannot be ruled out by clinical diagnosis. Positive mNGS results for some pathogens were confirmed by PCR or other methods using the remaining samples (see Table S2 in the supplemental material).

### Diagnostic assessment.

Definite infectious or noninfectious diseases were diagnosed by the combination of clinical characteristics, mNGS results, conventional detection, and treatment responses. Taking conventional methods as the gold standard, we calculated the sensitivity and specificity of mNGS. The total coincidence rate (TCR) of mNGS was evaluated based on the final clinical diagnosis. Positive and negative coincidence rates were defined as the coincidence rates against the final diagnosis for cases (or samples) that were positive and negative by mNGS, respectively.

### Statistical analysis.

Counts and percentages are presented for independent binomial variables. Means ± standard errors (SE) were calculated for continuous variables with normal distributions, while medians and interquartile ranges (IQRs) were used for abnormal distributions. Confidence intervals (CIs) were calculated according to the formula CI = average ± 1.96SE. A *t* test was performed to evaluate categorical variables, and a *P* value of <0.05 was considered statistically significant. The data were analyzed using IBM SPSS 25.0 and R 4.1.1.

### Data availability.

Sequencing data were deposited at the National Genomics Data Center under accession numbers PRJCA008034 and PRJCA008503. We declare that the main data supporting the findings are available within this article and the supplemental material. The other data generated and analyzed for this study are available from the corresponding author upon reasonable request.
