# Supplementary material for: Metagenomic Next-Generation Sequencing for the Diagnosis of Neonatal Infectious Diseases
Source: Microbiol Spectr. 2022 Nov 21;10(6):e01195-22. doi: 10.1128/spectrum.01195-22 (PMC9769891; doi:10.1128/spectrum.01195-22)
Supplement: Supplemental file 1 — Fig. S1 and S2 and Tables S1 to S3. Download spectrum.01195-22-s0001.pdf, PDF file, 0.7 MB [file spectrum.01195-22-s0001.pdf]

Metagenomic Next-Generation Sequencing for the Diagnosis of Neonatal Infectious Diseases

Lu Chen[1], Yujuan Zhao[1,*], Jiakai Wei[1], Wendi Huang[1], Ying Ma[1], Xuefeng Yang[2], Yang Liu[1], Jing Wang PhD[3], Han Xia[3,*], Zheng Lou PhD[3,*]

[1]Xi'an Children's Hospital, Xi'an, China

[2]Neonatal Intensive Care Department, Xi'an Children's Hospital, Xi'an, China

[3]Department of Scientific Affairs, Hugobiotech Co., Ltd., Beijing, China

*Correspondence: Y.Z. (zhaoyujuan69@163.com), H.X. (xiahan@hugobiotech.com), and Z.L. (louzheng@hugobiotech.com).

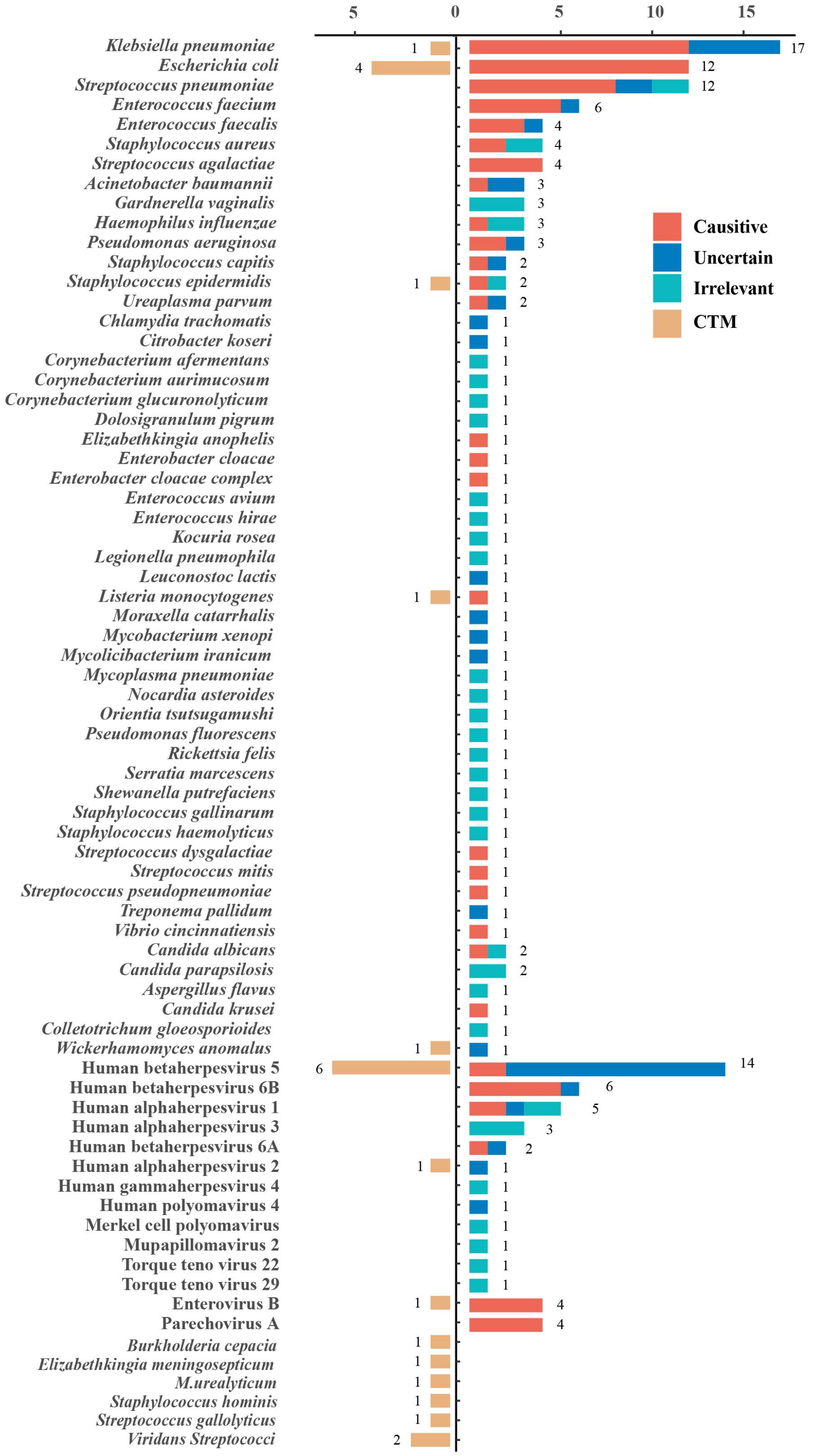

Figure S1 Pathogen profiles identified by mNGS and conventional methods (CTM) in 168 neonatal patients. Causative pathogens: the microbes identified by mNGS which can be confirmed by clinical diagnosis; Uncertain pathogens: the microbes identified by mNGS which cannot be proved

as causal agents but also cannot be ruled out by clinical diagnosis; Irrelevant pathogens: the microbes identified by mNGS which were not supported by clinical diagnosis. The number beside bar chart represents the number of neonatal patients with the pathogen.

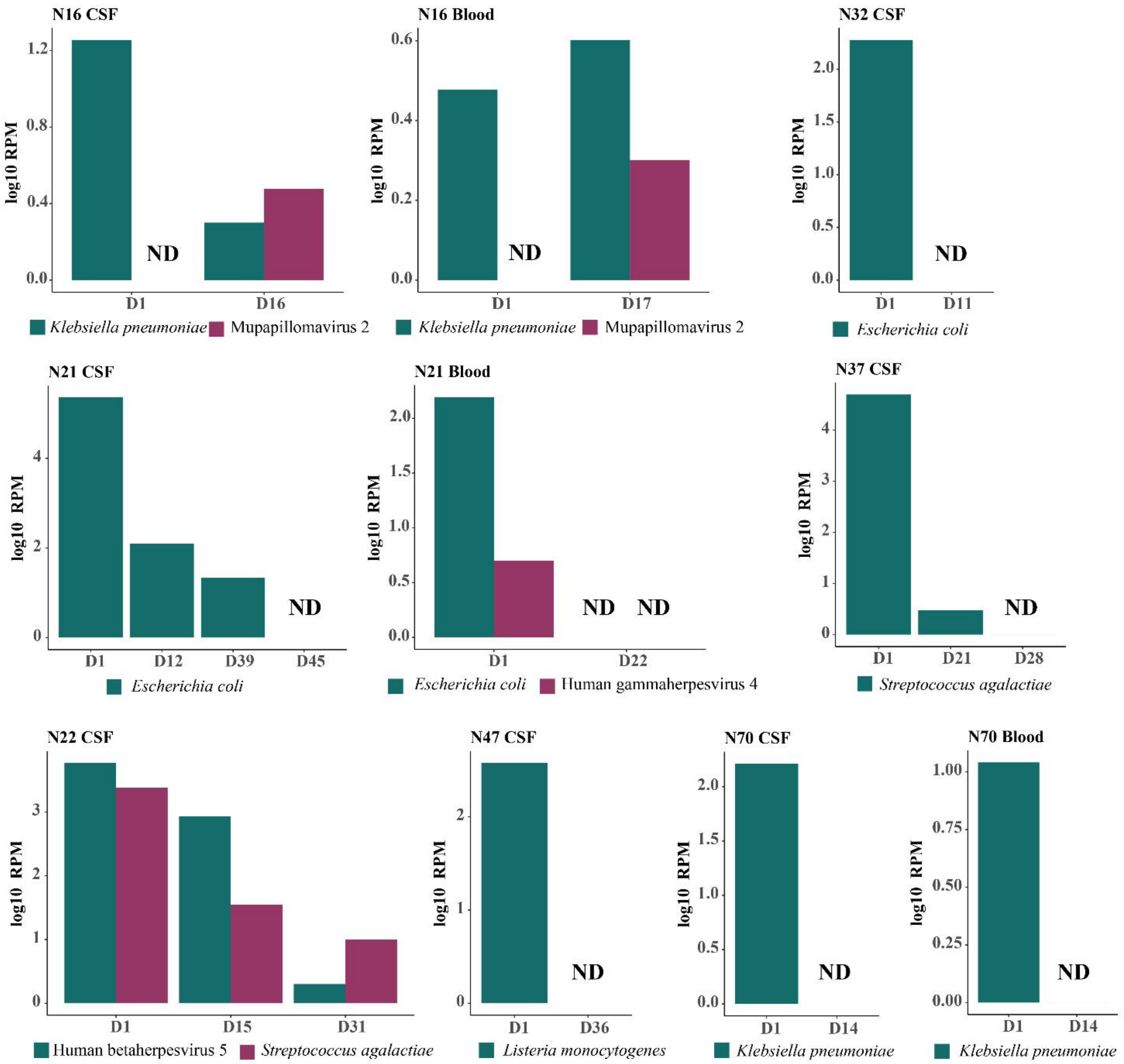

Figure S2 RPM change of 7 critically ill neonatal patients after adjusting treatment regimens 'ND': not detected.

Table S1 Clinical diagnoses of the participants

Infection		Case number
Central nervous system (CNS) infection		7
Bloodstream infection		29
Respiratory infection		15
Other infection		5
Co-infection		104
	CNS-Bloodstream	41
	CNS-Bloodstream-Respiratory tract	9
	CNS-Bloodstream-Respiratory tract-Other	2
	CNS-Other	1
	Bloodstream-Respiratory tract	30
	Bloodstream-Respiratory tract- Urinary tract	1
	Bloodstream-Respiratory tract-Other	2
	Bloodstream-Urinary tract	8
	Bloodstream-Other	6
	Respiratory tract-Other	2
Non-infection		10

Table S2 Confirmatory Testing

Patients	Samples	Pathogens detected by mNGS	Type of pathogens	Reads per million(RPM)	Confirmatory Testing
N22	Cerebrospinal fluid(CSF)	Human betaherpesvirus 5	Viruses	5875	qPCR assay (+, CT: 34.10)
N29	Blood	Human betaherpesvirus 5	Viruses	472	qPCR assay (+, CT: 32.50)
N50	Blood	Human betaherpesvirus 5	Viruses	60	qPCR assay (+, CT: 34.29)
N83	Blood	Human betaherpesvirus 5	Viruses	28	qPCR assay (+, CT: 36.99)
N92	Blood	Human betaherpesvirus 5	Viruses	298	qPCR assay (+, CT: 34.04)
N118	CSF	Human betaherpesvirus 5	Viruses	5	qPCR assay (-)
N118	Blood	Human betaherpesvirus 5	Viruses	355	qPCR assay (+, CT: 34.01)
N122	Blood	Human betaherpesvirus 5	Viruses	1338	qPCR assay (+, CT: 32.55)
N93	Blood	Human betaherpesvirus 5	Viruses	173	insufficient sample
N47	CSF	*Listeria monocytogenes*	Bacterium	377	PCR assay (+)
N1	CSF	*Candida albicans*	Fungus	256	PCR assay (+)
N21	CSF	*Escherichia coli*	Bacterium	228289	PCR assay (+), confirmed by Sanger sequencing
N21	Blood	*Escherichia coli*	Bacterium	155	PCR assay (+), confirmed by Sanger sequencing
N21	CSF	*Escherichia coli*	Bacterium	125	PCR assay (-)
N32	CSF	*Escherichia coli*	Bacterium	189	PCR assay (+), confirmed by Sanger sequencing
N69	CSF	*Escherichia coli*	Bacterium	3852	PCR assay (+), confirmed by Sanger sequencing
N87	Blood	*Escherichia coli*	Bacterium	203	PCR assay (-)
N123	Blood	*Escherichia coli*	Bacterium	781	PCR assay (+), confirmed by Sanger sequencing
N22	CSF	*Streptococcus agalactiae*	Bacterium	2413	PCR assay (+), confirmed by Sanger

					sequencing
N37	CSF	*Streptococcus agalactiae*	Bacterium	49696	PCR assay (+), confirmed by Sanger sequencing
N78	Blood	Human betaherpesvirus 6B	Viruses	10176	PCR assay (+), confirmed by Sanger sequencing
N106	Blood	Human betaherpesvirus 6A	Viruses	423	PCR assay (+), confirmed by Sanger sequencing

Table S3 Microbes identified from neonatal patients with blood-CNS co-infection by mNGS of both blood and cerebrospinal fluid (CSF) samples

Patients	Nucleic acid testing	Samples	Pathogens detected by mNGS	Type of pathogens	Coincidence or Non-coincidence with final diagnosis
N2	DNA	CSF	Human alphaherpesvirus 2	Virus	Non-coincidence
	DNA	Blood	Human alphaherpesvirus 2	Virus	
N7	DNA	CSF	*Klebsiella pneumoniae*	Bacterium	Non-coincidence
			Human alphaherpesvirus 3	Virus	
	DNA	Blood	Human alphaherpesvirus 3	Virus	
N10	DNA	CSF	*Klebsiella pneumoniae*	Bacterium	Coincidence
	DNA	Blood	*Klebsiella pneumoniae*	Bacterium	
N14	DNA	CSF	*Gardnerella vaginalis*	Bacterium	Non-coincidence
	DNA	Blood	Human alphaherpesvirus 3	Virus	
			Haemophilus influenzae	Bacterium	
N16	DNA	Blood	*Klebsiella pneumoniae*	Bacterium	Coincidence
	DNA	CSF	*Klebsiella pneumoniae*	Bacterium	
N20	DNA	CSF	*Corynebacterium glucuronolyticum*	Bacterium	Non-coincidence
			Mycolicibacterium iranicum	Bacterium	
	DNA	Blood	*Mycolicibacterium iranicum*	Bacterium	
N21	DNA	CSF	*Escherichia coli*	Bacterium	Coincidence
	DNA	Blood	*Escherichia coli*	Bacterium	
			Human gammaherpesvirus 4	Virus	
N23	DNA	CSF	*Serratia marcescens*	Bacterium	Non-coincidence
	DNA	Blood	*Chlamydia trachomatis*	Chlamydia	
N69	DNA	CSF	*Escherichia coli*	Bacterium	Coincidence
			Citrobacter koseri	Bacterium	
	DNA	Blood	*Escherichia coli*	Bacterium	
N70	DNA	CSF	*Klebsiella pneumoniae*	Bacterium	Coincidence
	DNA	Blood	*Klebsiella pneumoniae*	Bacterium	
N82	RNA	CSF	Parechovirus A	Virus	Coincidence
	RNA	Blood	Parechovirus A	Virus	
N84	DNA	CSF	*Elizabethkingia anophelis*	Bacterium	Coincidence
	DNA	Blood	Human betaherpesvirus 5	Virus	Non-coincidence
N95	RNA	Blood	Parechovirus A	Virus	Coincidence
	RNA	CSF	Parechovirus A	Virus	
N105	DNA	Blood	*Acinetobacter baumannii*	Bacterium	Non-coincidence
	DNA	CSF	*Staphylococcus aureus*	Bacterium	Coincidence
N106	DNA	CSF	Human betaherpesvirus 6A	Virus	Coincidence
	DNA	Blood	Human betaherpesvirus 6A	Virus	
N8	DNA	CSF	*Klebsiella pneumoniae*	Bacterium	Non-coincidence

			Streptococcus pneumoniae	Bacterium	
	DNA	Blood	/	/	
N47	DNA	CSF	*Listeria monocytogenes*	Bacterium	Coincidence
	DNA	Blood	/	/	Non-coincidence
N58	DNA	CSF	*Streptococcus agalactiae*	Bacterium	Coincidence
	DNA	Blood	/	/	Non-coincidence
N60	DNA	CSF	*Escherichia coli*	Bacterium	Coincidence
	DNA	Blood	/	/	Non-coincidence
N100	DNA	CSF	*Streptococcus mitis*	Bacterium	Coincidence
	DNA	Blood	/	/	Non-coincidence
N19	DNA	CSF	/	/	Non-coincidence
	DNA	Blood	*Gardnerella vaginalis*<br>*Aspergillus flavus*	Bacterium<br>Fungus	
N30	DNA	CSF	/	/	Non-coincidence
	DNA	Blood	*Staphylococcus capitis*	Bacterium	
N59	DNA	CSF	/	/	Non-coincidence
	DNA	Blood	*Pseudomonas aeruginosa*	Bacterium	Coincidence
N147	DNA	CSF	/	/	Non-coincidence
	DNA	Blood	Human betaherpesvirus 5<br>*Haemophilus influenzae*	Virus<br>Bacterium	Coincidence
N43	DNA	CSF	/	/	Non-coincidence
	DNA	Blood	/	/	
N49	DNA	CSF	/	/	Non-coincidence
	DNA	Blood	/	/	
N64	DNA	CSF	/	/	Non-coincidence
	DNA	Blood	/	/	
